# Comparison of Large Language Model with Aphasia

**DOI:** 10.1002/advs.202414016

**Published:** 2025-05-14

**Authors:** Takamitsu Watanabe, Katsuma Inoue, Yasuo Kuniyoshi, Kohei Nakajima, Kazuyuki Aihara

**Affiliations:** ^1^ International Research Centre for Neurointelligence The University of Tokyo Institutes for Advanced Study 7‐3‐1 Hongo Bunkyo‐ku Tokyo 113‐0033 Japan; ^2^ Graduate School of Information Science and Technology The University of Tokyo Tokyo 113‐8656 Japan

**Keywords:** aphasia, energy landscape analysis, large language model

## Abstract

Large language models (LLMs) respond fluently but often inaccurately, which resembles aphasia in humans. Does this behavioral similarity indicate any resemblance in internal information processing between LLMs and aphasic humans? Here, we address this question by comparing the network dynamics between LLMs—ALBERT, GPT‐2, Llama‐3.1 and one Japanese variant of Llama—and various aphasic brains. Using energy landscape analysis, we quantify how frequently the network activity pattern is likely to move from one state to another (transition frequency) and how long it tends to dwell in each state (dwelling time). First, by investigating the frequency spectrums of these two indices for brain dynamics, we find that the degrees of the polarization of the transition frequency and dwelling time enable accurate classification of receptive aphasia, expressive aphasia and controls: receptive aphasia shows the bimodal distributions for both indices, whereas expressive aphasia exhibits the most uniform distributions. In parallel, we identify highly polarized distributions in both transition frequency and dwelling time in the network dynamics in the four LLMs. These findings indicate the similarity in internal information processing between LLMs and receptive aphasia, and the current approach can provide a novel diagnosis and classification tool for LLMs and help their performance improve.

## Graphical abstract

Large language models (LLMs) answer almost all questions fluently but often inaccurately, which resembles a specific type of aphasia in humans. Using a data‐driven analysis called energy landscape analysis, this study reveals similarities in the internal information dynamics between LLMs and the brains of humans with receptive aphasia, such as Wernicke's aphasia.

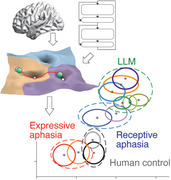

## 1 Introduction

Major large language models (LLMs) fluently generate persuasive answers to almost all inquiries,^[^ ^1^ ^]^ but their responses are often inaccurate^[^ ^2^, ^3^, ^4^ ^]^ and sometimes contain hallucinations.^[^ ^5^, ^6^ ^]^ This behavioral tendency resembles symptoms of receptive aphasia in humans: for example, individuals with Wernicke's aphasia—a representative type of receptive aphasia—are able to speak with normal rhythm and grammatical accuracy, but their speech content often does not make sense.^[^ ^7^ ^]^ Also, repetition of noninformative words is observed among the symptoms of Wernicke's aphasia^[^ ^7^ ^]^ and in hallucinated outputs of LLMs as well.^[^ ^6^, ^8^ ^]^ A recent report on LLMs’ insensitivity to underlying messages^[^ ^8^ ^]^ would be comparable to the difficulty in language comprehension seen in receptive aphasia.^[^ ^7^, ^9^ ^]^ Furthermore, we can argue linguistic similarities between certain types of aphasia and LLMs. First, despite their recent development, LLMs’ outputs show significant structural differences from human‐written texts^[^ ^4^, ^10^ ^]^ and still cannot produce as much linguistic diversity and variation as human writers can.^[^ ^11^ ^]^ Conversely, the abrupt and sudden topic shifts often seen in their hallucinated outputs^[^ ^12^ ^]^ are similar to the decrease in the linguistic cohesion observed in the speech of individuals with receptive aphasia.^[^ ^13^ ^]^

These behavioral and linguistic resemblances between LLMs and aphasia—especially, receptive aphasia—in humans may indicate similarities even in internal information processing between them. Here, to examine this indication, we compared the dynamics of network activity observed in LLMs with the collective neural dynamics seen in human brains with various aphasia. We focused on such network dynamics since a line of human neuroimaging studies suggested that collective neural dynamics are closely linked with one's cognitive ability and intelligence.^[^ ^14^, ^15^, ^16^ ^]^

**Figure 1:**
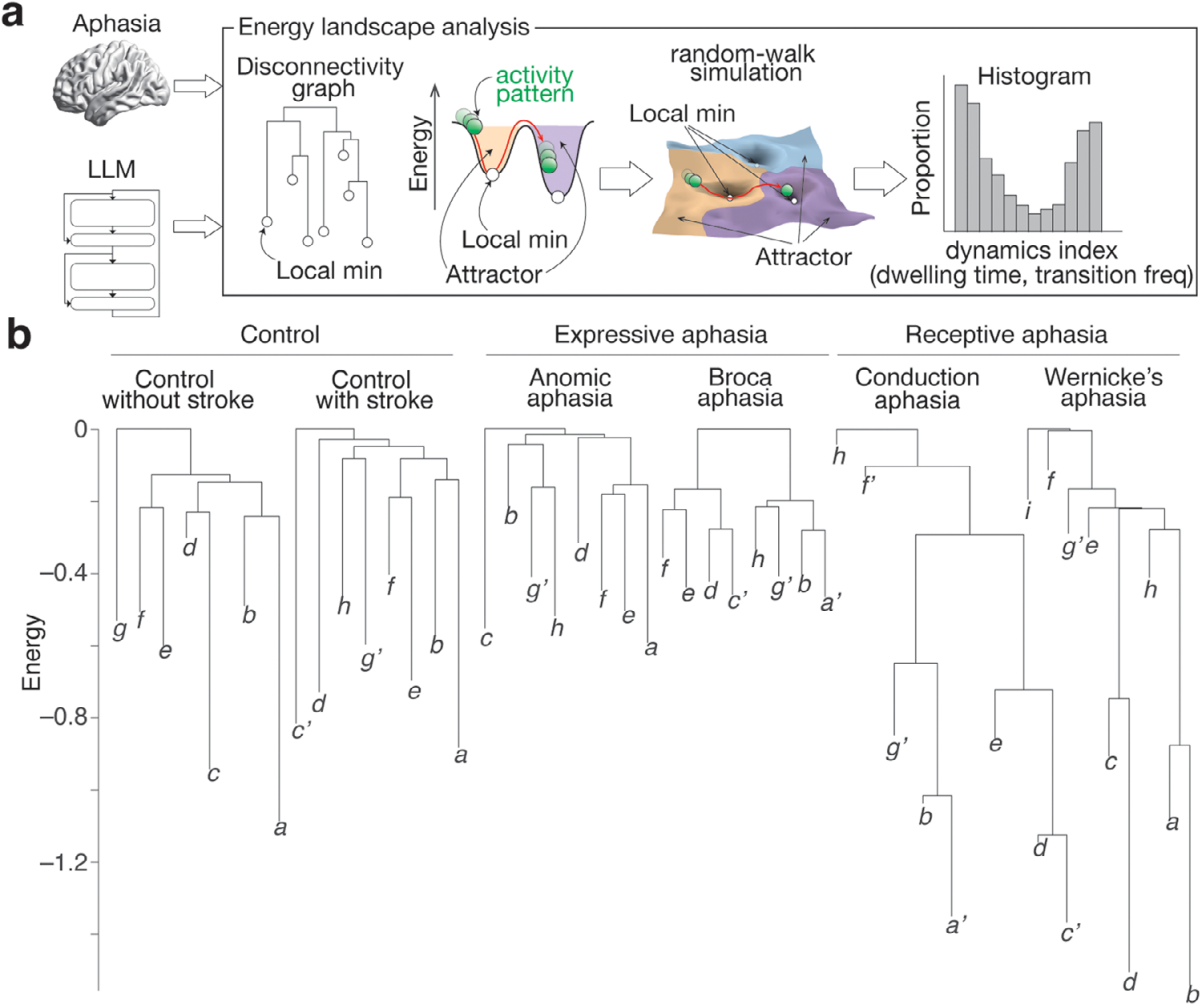
Energy landscape analysis. a) Using energy landscape analysis, we compared the network dynamics between aphasic human brains and alarge language models (LLMs). For the aphasic brain dynamics, we analyzed resting‐state functional MRI data of each individual and identified each energy landscape with multiple local minima. Then, by conducting a random‐walk simulation on the energy landscape, we calculated the dwelling time for each local minimum and transition frequency between every pair of the local minima. We applied the same analysis to the internal network activity occurring in LLMs. **b**. For presentation purposes, the six dendrograms—so‐called dysconnectivity graphs—show the results of the group‐level energy landscape structures for the six different groups. The same letters indicate the same local minima, whereas the apostrophised letters denote similar local minima (activation similarity ≥89%). Note that the main analyses were performed at an individual level.

Collective network dynamics were assessed with energy landscape analysis,^[^ ^17^, ^18^ ^]^ which enables us to depict dynamic changes occurring in a network as a ball movement among different attractors that are defined as local minima on a hypothetical energy landscape (**Figure** **1**a; see Experimental Section for details). We chose this method since it can extract such network dynamics from various types of time‐series data, including not only neural signals of human brains^[^ ^18^, ^19^, ^20^, ^21^ ^]^ but also activities of microbiota in rodents’ guts.^[^ ^22^ ^]^ In particular, regarding human brains, this data‐driven approach has successfully detected atypical brain dynamics underlying a wide range of neuropsychiatric conditions, such as autism,^[^ ^16^, ^23^ ^]^ ADHD,^[^ ^23^, ^24^ ^]^ depression,^[^ ^25^ ^]^ Schizophrenia,^[^ ^26^ ^]^ epilepsy^[^ ^27^ ^]^ and Alzheimer's disease.^[^ ^28^ ^]^

To identify the intrinsic brain dynamics underlying aphasia, we analyzed resting‐state fMRI (rsfMRI) data recorded from stroke patients with four different types of aphasia, stroke patients without any aphasia, and healthy individuals without any brain stroke nor aphasia symptoms (**Table** **1**). We examined the resting‐state brain signals since this neural activity, which was collected without asking participants to engage in any specific cognitive task, is known to represent the functional backbones of a wide range of cognitive functions.^[^ ^14^, ^29^, ^30^, ^31^, ^32^ ^]^ In fact, analyzing the rsfMRI data revealed fundamental neural characteristics underlying various neuropsychiatric disorders.^[^ ^16^, ^33^, ^34^, ^35^, ^36^ ^]^

**Table 1:** Demographic data for aphasic individuals.

		Individuals with stroke				
	Control without stroke	Control	Anomic aphasia	Broca aphasia	Conduction	Wernicke's
*N*	63	15	52	130	46	6
Age (mean±sd)	63.3 ± 3.8	64.5 ± 6.6	67.4 ± 6.7	63.7 ± 7.7	60.4 ± 9.0	59.2 ± 2.4
Comparison with control with stroke	0.8	–	0.1	0.6	0.1	0.1

To investigate network dynamics occurring in LLMs, we used four LLMs: A‐Lite Bidirectional Encoder Representations from Transformers (ALBERT),^[^ ^37^ ^]^ an LLM developed by Google; GPT‐2^[^ ^38^ ^]^ by OpenAI; Llama‐3.1^[^ ^39^ ^]^ by Meta; LLM‐jp‐3,^[^ ^40^ ^]^ a Japanese variant of Llama developed by National Institute of Informatics (NII) in Japan.

We chose these LLMs since they share the same principle with other representative LLMs, such as GPT 4.^[^ ^41^ ^]^ That is, they are built upon the attention mechanism^[^ ^42^ ^]^—a building block of the Transformer—and generate internal dynamics by iterating this structure^[^ ^43^ ^]^ (see Experimental Section for the definition of ‘internal dynamics’ in LLMs). In addition, the four LLMs were practical choices because their codes are publicly available and small enough for us to handle in laboratory settings.

Furthermore, we selected these LLMs because such LLMs are considered to share computational principles of language processing with the human brains.^[^ ^44^, ^45^, ^46^ ^]^ In particular, several studies reported evidence for the LLM‐brain similarity for GPT‐2: one research showed that GPT‐2 can be a good model to understand how humans predict the next words and comprehend a stream of languages;^[^ ^47^ ^]^ another study showed significant similarities between human neural activity during language comprehension and information processing occurring in GPT‐2.^[^ ^48^ ^]^ These findings indicate that it is reasonable to compare how these LLMs compute language information with how the human brains comprehend and generate language.

Given these, we adopted the four LLMs and compared their internal dynamics with those seen in the human brains.

Note that, for comparisons with the resting‐state brain dynamics in humans, we analyzed the internal network activity that occurred in the LLMs after the LLM generated an answer to the input (Figure S1, Supporting Information). Such an LLM network activity can be regarded as a representation of a quasi‐static state of the LLM's information processing, which we assume is comparable to resting‐state brain dynamics.^[^ ^49^, ^50^ ^]^

## 2 Results

### 2.1 Brain Dynamics of Aphasic Humans

We first characterized the aphasic brain dynamics. After confirming that a pairwise maximum entropy model—a basis of energy landscape analysis—was accurately fitted to all the individual rsfMRI data (accuracy >90.1%), we constructed an energy landscape for each participant and found different energy landscape structures in different types of aphasia (see Figure 1b for the group‐level results as examples). Compared to the controls, the brains with expressive aphasia exhibited shallower and more uniform attractors, whereas the brains with receptive aphasia contained both deep and shallow attractors.

**Figure 2:**
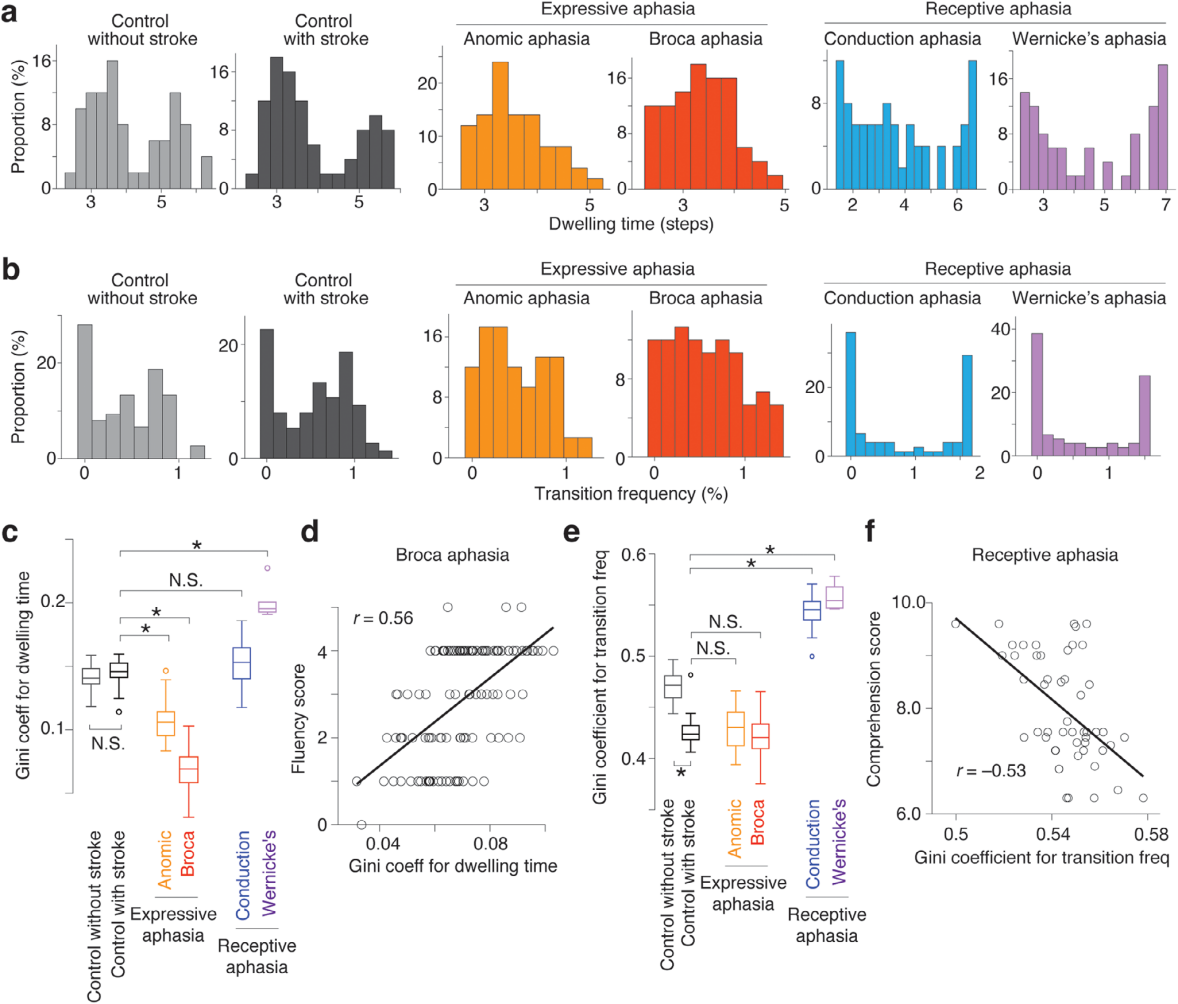
**Brain dynamics of aphasia. a and b**. For each group, we assessed the distributions of the dwelling time in each attractor (**Panel a**) and the transition frequency among different attractors (**Panel b**). **c‐f**. We then calculated the degrees of the polarization of these distributions by estimating Gini coefficients for dwelling time and transition frequency, respectively, at an individual level. Compared to the control individuals with stroke, the individuals with expressive aphasia had significantly smaller Gini coefficients (**Panel c**). The reduced Gini coefficients seen in the individuals with Broca aphasia were correlated with their impairment of speech fluency (**Panel d**). As to transition frequency, the Gini coefficient was significantly larger in the receptive aphasia than in the controls (**Panel e**), and such an atypically large Gini coefficient observed in the receptive aphasia was correlated with its impairment of comprehension ability (**Panel f**). In Panels d and f, each circle represents each individual. ^*^ *P*_Bonferroni_ < 0.05.

We then performed a random‐walk simulation on each energy landscape and quantified brain dynamics for each group. Specifically, we calculated how long the brain activity pattern stayed at each attractor (dwelling time; **Figure** **2**a) and how often it transited among different attractors (transition frequency; Figure 2b). As a result, we found that, in the expressive aphasia, the energy landscape with shallow and uniform attractors tended to yield relatively uniform distributions for both the dwelling time and transition frequency. In contrast, in the receptive aphasia, the energy landscape consisting of attractors with diverse depths was likely to generate bimodal and polarized distributions of the two brain dynamics indices.

### 2.2 Characterization of Aphasia based on Gini Coefficients

This qualitative difference between the different types of aphasia was quantitatively confirmed by assessing Gini coefficients for the dwelling time distributions and transition frequency distributions. We calculated the Gini coefficient since the index can quantify the degree of the polarization of a distribution. For example, if the transition frequency showed a bimodal distribution, the Gini coefficient for the transition frequency should be close to 1. If the metric for the neural dynamics exhibited a uniform distribution, its Gini coefficient should be close to 0.

First, in the expressive aphasia, we found significantly lower Gini coefficients for the dwelling time than those in the controls with stroke (*t* > 7.3, *P*_Bonferroni_ <0.05, *η^2^* > 0.33; Figure 2c). Additionally, this aberrant decrease in the Gini coefficient was correlated with the damaged speech fluency observed in Broca aphasia (*r*_128_ = 0.56, *p* < 10^−5^; Figure 2d). In contrast, the Gini coefficients for the transition frequency did not significantly differ from those in the control group (*t* < 1.3, *p* > 0.16; Figure 2e).

In the receptive aphasia group, we identified the opposite pattern: the Gini coefficients for the dwelling time seen in this aphasia group showed only marginal differences from those in the control groups (*t* <1.9, *p* > 0.06 for conduction aphasia; Figure 2c); in contrast, their Gini coefficients for the transition frequency were significantly larger than those in the controls (*t* > 10.9, *P*_Bonferroni_ <0.05, *η^2^* > 0.67; Figure 2e), and this atypically high Gini coefficient was correlated with the poor comprehension capability seen in the receptive aphasia (*r*_49_ = −0.53, *p* < 10^−5^; Figure 2f).

**Figure 3:**
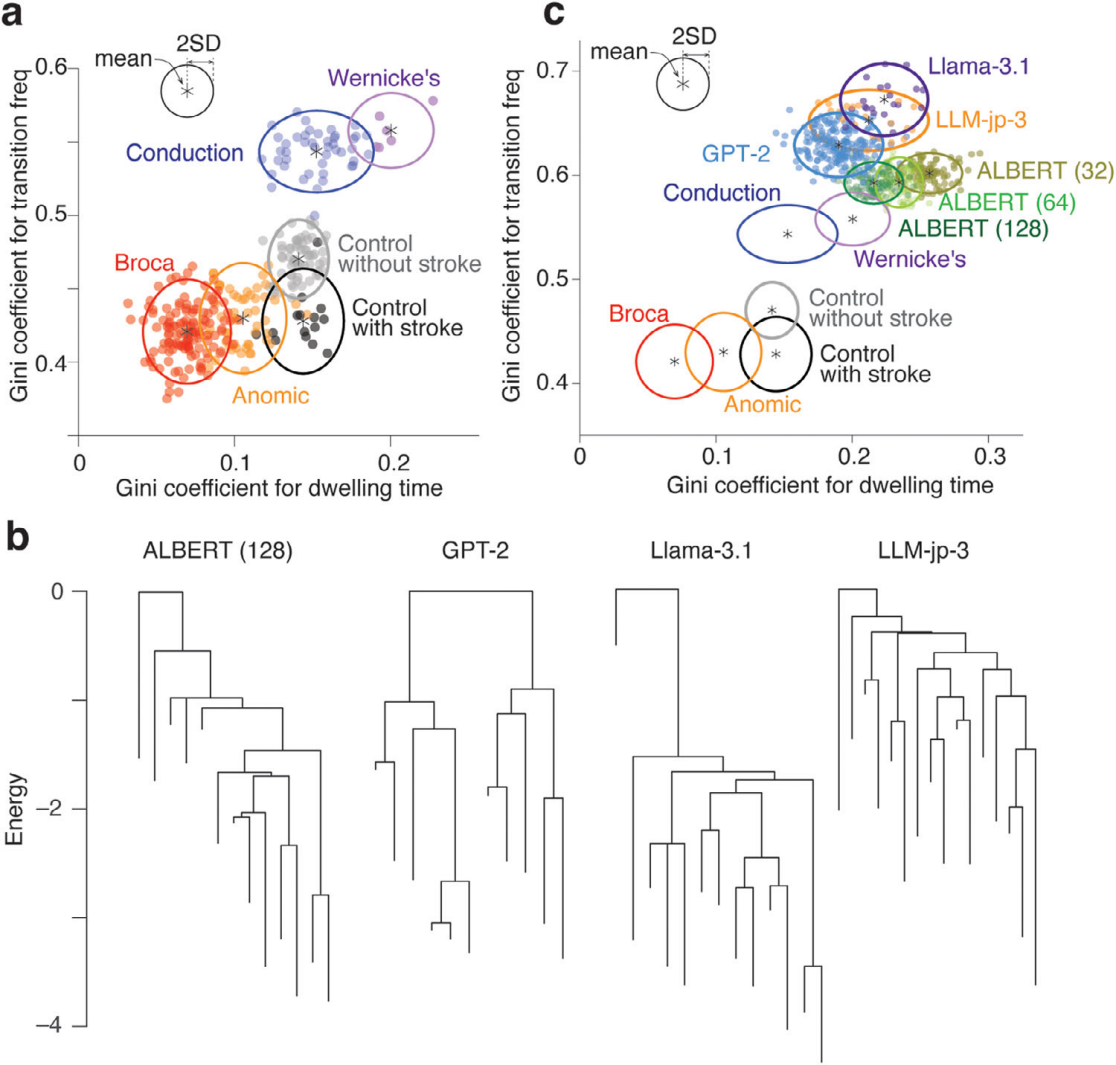
**Comparison between aphasia and LLMs. a**. Based on the two Gini coefficients (one for transition frequency and the other for dwelling time), we could distinguish between expressive aphasia (anomic and Broca aphasia), receptive aphasia (conduction and Wernicke's aphasia), and controls relatively clearly. Each circle represents each individual. **b** We applied energy landscape analysis to network activities recorded from the LLMs (ALBERT, GPT‐2, Llama‐3.1 and LLM‐jp‐3). The dendrogram shows examples of the energy landscape structures for the four LLMs. **c**. We then calculated the two types of Gini coefficient for the LLMs and found that the network dynamics indices for the four LLMs were located close to those for Wernicke's aphasia.

These results suggest that the two Gini coefficients for the dwelling time and transition frequency could be indices to classify the types of aphasia based on the internal network dynamics (**Figure** **3**a).

### 2.3 Application to LLMs

Given these findings, we then compared internal network dynamics between aphasia and LLMs. Specifically, using energy landscape analysis, we first examined internal network activity recorded from the four LLMs (ALBERT, GPT‐2, Llama‐3.1 and LLM‐jp‐3). We then calculated their Gini coefficients for the dwelling time and transition frequency, and finally compared the two indices with those observed in the aphasia. For Google ALBERT, we used three different internal network activity data recorded from the LLM, which processed three different lengths of tokens (i.e., 32, 64 and 128). The other LLMs were given 20‐length tokens.

First, we found that, in all the LLMs, the energy landscapes had deeper attractors than the human brains (Figure 3b). We then calculated Gini coefficients for both the dwelling time and transition frequency (Figure 3c) and revealed that the two Gini coefficients for the LLMs were significantly larger than those seen in the human controls (*t* > 6.6, *P*_Bonferroni_ <0.05, *η^2^* > 0.17 for dwelling time; *t* > 9.3, *P*_Bonferroni_ <0.05, *η^2^* > 0.28 for transition frequency) and the expressive aphasia group (*t* > 22.1, *P*_Bonferroni_ < 0.05, *η^2^* > 0.69 for dwelling time; *t* > 25.4, *P*_Bonferroni_ < 0.05, *η^2^* > 0.75 for transition frequency). In contrast, such distinct differences were not seen between the LLMs and the receptive aphasia (e.g., *t* = 1.5, *P*_Bonferroni_ > 0.05 for a two‐sample *t*‐test of dwelling time between the GPT 2 and Wernicke's aphasia).

Taken together, these results suggest that the internal network dynamics seen in ALBERT, GPT‐2, Llama‐3.1 and LLM‐jp‐3 were more similar to collective brain dynamics underlying the receptive aphasia than to those seen in the expressive aphasia or controls.

## 3 Discussion

Inspired by the behavioral resemblance between aphasia and LLMs, we investigated similarities in the internal information processing between them. As a preparation, we analyzed the collective neural dynamics underlying expressive and receptive aphasia using energy landscape analysis and found that the degrees of polarization of the transition frequency and dwelling time on the hypothetical energy surface are the key to brain‐dynamics‐based classification of aphasia. We then applied this result to LLMs and identified a significant similarity between internal dynamics in the four LLMs (ALBERT, GPT‐2, Llama‐3.1 and LLM‐jp‐3) and collective brain dynamics seen in receptive aphasia.

One of the limitations of the current study is the number of types of aphasia and LLMs we investigated here. Regarding aphasia, we analyzed brain dynamics observed in four representative aphasia (Table 1); but, due to the difficulty of data availability, we did not examine other relatively rare types of this disorder, such as transcortical motor aphasia and transcortical sensory aphasia. In addition, the sample sizes of some aphasic individuals are relatively small. Given these, future studies would have to test the generalizability of the current observations with larger and more diverse samples.

As to LLMs, the current study investigated ALBERT, GPT‐2, Llama‐3.1, and LLM‐jp‐3 as examples of LLMs mainly because they have internal dynamics to which energy landscape analysis can be applied. In the meantime, the network structure of this LLM is relatively simple (e.g., 235 million parameters for the xxlarge model in ALBERT) compared with other LLMs such as GPT 3,^[^ ^51^ ^]^ which has 175 billion parameters. Considering this, we would have to examine network dynamics in larger LLMs in future studies.

Another limitation is that we did not investigate the biological mechanisms underlying the aphasia. This study focused on the meta‐comparison between the human brain dynamics and *in‐silico* phenomena occurring inside the LLMs. A line of human neuroimaging studies have reported aphasia‐specific atypical neural activities and functional and structural abnormalities of large‐scale brain networks.^[^ ^9^, ^52^, ^53^, ^54^, ^55^, ^56^, ^57^, ^58^, ^59^, ^60^ ^]^ The current observations on the brain state dynamics underlying a variety of aphasia would be expected to contribute to further neurobiological understanding of this disorder; but, to achieve that, it would be necessary to conduct a more comprehensive neuroscientific investigation on the atypical brain dynamics we found here.

In addition, we have to mention the possibility that the inaccurate responses of the LLMs and their so‐called hallucinations may not be due to the same causes as those underpinning the symptoms of receptive aphasia in humans. Although the exact mechanisms that generate hallucinations are unidentified, such incorrect but highly fluent responses often seen in LLMs are considered to be related to inaccurate knowledge acquired during their pretraining.^[^ ^61^ ^]^ In fact, the improvement of the quality of the pretraining is known to lead to less hallucinated outputs in LLMs.^[^ ^62^, ^63^ ^]^ In other words, inaccurate data for the pretraining would increase the shallow language matching inside LLMs, resulting in their hallucinated responses. In contrast, receptive aphasia in humans is reported to be associated with the stroke‐induced damages to the appropriate matchings of words and sentences that are thought to be implemented in brain‐wide networks.^[^ ^64^, ^65^ ^]^

In particular, for Wernicke's aphasia, we also have to clarify behavioral and linguistic differences between its symptoms and typical hallucinations generated by LLMs. Individuals with Wernicke's aphasia have difficulty in language comprehension but can speak fluently and tend to repeat noninformative words,^[^ ^7^ ^]^ all of which are often observed in LLMs.^[^ ^6^, ^8^ ^]^ However, unlike LLMs, the speech seen in Wernicke's aphasia is likely to show less linguistic cohesion^[^ ^13^ ^]^ and complexity.^[^ ^66^, ^67^ ^]^ In fact, it is known that some LLMs, such as GPT‐4, can improve linguistic cohesion of essays written by humans.^[^ ^68^ ^]^

Given these, future studies would have to clarify not only the similarities but the differences between hallucinations in LLMs and symptoms of receptive aphasia by scrutinizing the details of their information processing and its resulting linguistic behaviors.

Despite these limitations, the current study may present a new concept on the relationship between human brains and LLMs. With the rapid development of LLMs, it is becoming intensively difficult to understand their internal information processing.^[^ ^69^ ^]^ As a result, most evaluations of the LLMs are likely to be performed by examining their outputs.^[^ ^51^, ^70^ ^]^ In contrast, the current study has pointed out a possibility that we may be able to give a certain diagnosis to an LLM by comparing its internal network dynamics with typical/atypical human brain dynamics. This approach may lead to developing a novel methodology to characterize LLMs, which would enable us to infer their performance even before using them and looking into their outputs.

## 4 Experimental Section

### Overall Design

We compared network dynamics between aphasic brains and LLMs using energy landscape analysis. As for aphasia, we analyzed resting‐state fMRI data obtained from i) individuals who had aphasia due to brain stroke, ii) those who had brain stroke but no aphasic symptoms, and iii) those who had neither stroke nor aphasia. As to the LLMs, we collected internal dynamics from ALBERT, GPT‐2, Llama‐3.1, and LLM‐jp‐3 after we put some random inputs into the LLMs. Then, we applied energy landscape analysis to the data and quantified the network dynamics.

### Analysis of the Human Brain Data

*MRI Data*: To investigate brain dynamics in aphasia, we analyzed rsfMRI data shared in two datasets: the Aphasia Recovery Cohort (ARC) dataset^[^ ^71^ ^]^ and the PREVENT‐AD project.^[^ ^72^ ^]^ The ARC dataset was collected from individuals who had different types of aphasia due to brain stroke and those with stroke but no aphasic symptoms. The MRI data were recorded at the University of South Carolina using multiple 3.0T MRI scanners (TrioTim/Prisma Fit, Siemens Medical Systems). T1‐weighted anatomical images were collected at 1mm‐cubic resolution. Functional images were recorded using an echo planar imaging (EPI) sequence (TR 2sec, TE 30ms, Flip angle 90°, 36 slices, spatial resolution 3×3×3mm). Phase image and magnitude images were also obtained for a fieldmap.

We used the PREVENT‐AD dataset to obtain MRI data from cognitively normal individuals who had neither stroke nor aphasia. The MRI data were recorded using a 3.0 T MRI scanner (Magnetom Tim Trio, Siemens Medical Systems). Functional images were collected using EPI sequence (TR 2sec, TE 30ms, Flip angle 90°, 32 slices, spatial resolution 4×4×4 mm). Anatomical images were obtained as T1*‐weighted MRI data with a 1mm‐cubic resolution.

*Ethics*: The recording of the ARC data was approved by the Institutional Review Board at the University of South Carolina. The acquisition of the PREVENT‐AD data was approved by the “Research, Ethics and Compliance Committee” of McGill University. In both the datasets, the participants gave informed consent in a written form.

*Participants*: For the ARC dataset, we focused on the data obtained from individuals with anomic/Broca/conduction/Wernicke's aphasia and age‐matched individuals with stroke but no aphasia. We used the PREVENT‐AD dataset to obtain the data of another control group without stroke.

*Preprocessing of Brain Data for Energy Landscape Analysis*: The EPI data were preprocessed with SPM12 (www.fil.ucl.ac.uk/spm) in the same way as in the previous studies using the energy landscape analysis for rsfMRI data.^[^ ^16^, ^23^ ^]^ First, the first five images were discarded, and the images underwent realignment, unwarping, slice timing correction, normalization to the standard template (ICBM 152) and spatial smoothing (Gaussian kernel with 8mm of full‐width at half maximum). Then, we removed the effects of head motion, white matter signals, cerebrospinal fluid signals and global signals before performing band‐pass temporal filtering (0.01–0.1Hz). No significant difference was found in any of the six parameters for the head motion between the control with strokes and the other groups (*p* > 0.4).

For the ARC datasets, we then used an individual anatomical mask image that was defined for each patient and indicated the brain lesion caused by stroke. After preprocessing the anatomical mask image, we overlayed the mask to the preprocessed rsfMRI images, set voxels in the stroke lesion as “Not a Number” (i.e., “NaN” in Matlab) and omitted the rsfMRI signals of the stoke lesion from the following analyses. We performed this processing for each individual.

To apply energy landscape analysis to this preprocessed rsfMRI dataset, we then divided the cerebral cortex into the following nine functionally distinct networks: the default mode network (DMN), frontoparietal network (FPN), salience network (SAN), dorsal attention network (DAN), ventral attention network (VAN), cingulo‐opercular network (CON), somatosensory network (SMN), auditory network (AN) and visual network (VN). We performed this functional segmentation based on a widely used brain parcellation system^[^ ^73^ ^]^ since the previous studies using the same parcellation system succeeded in capturing brain state dynamics specific to ASD,^[^ ^16^, ^23^ ^]^ ADHD^[^ ^23^ ^]^ and ASD+ADHD comorbidity.^[^ ^23^ ^]^

Technically, we extracted time‐series rsfMRI data from each region of interest, which was defined as a 4‐mm‐radius sphere around the pre‐determined MNI center coordinate, and assigned the data to the corresponding network based on the parcellation system.^[^ ^73^ ^]^ Next, we calculated the average rsfMRI signal for each network at each time point for each participant and then binarized the network activities using the whole‐brain average fMRI signal as a threshold (+1 for active and –1 for inactive). This binarization procedure balanced the numbers of active and inactive states and should improve the accuracy of the following analysis,^[^ ^17^ ^]^ which was also face‐validated in the previous work.^[^ ^15^, ^16^, ^19^, ^23^ ^]^ After this procedure, an activity pattern of the nine networks at time point *t* was described such as $V^{t}=\left[\sigma_{1}^{t},\sigma_{2}^{t},\text{…},\;\sigma_{N}^{t}\right]$, where $\sigma_{i}^{t}$ represented a binary activity of network *i* at time *t* (i.e., $\sigma_{i}^{t}=+1\;\mathrm{or}-1$) and *N* denotes the number of the networks (here, *N* = 9).

### LLM Data Generation

Most LLMs are based on the neural network architecture known as the Transformer,^[^ ^37^ ^]^ which comprises two components: an encoder and a decoder. Once raw input text is uniquely converted into a sequence of tokens—integers extracted from a finite set called *vocabulary V*—by a'tokenizer', the encoder transforms each token into a high‐dimensional continuous vector; this process is often referred to as *embedding*. In contrast, the decoder not only shares the same components as the encoder but also has an additional mechanism to generate the subsequent token. Notably, recent studies have proposed models that utilize only the encoder (encoder‐only model) or solely the decoder (decoder‐only model). Here, we focused on these two types of models and sampled the temporal evolution of the internal states from them. This study named such a series of internal states ‘internal dynamics’.

*Encoder‐Only Model*: Encoder‐only models are neural networks composed of attention blocks—each consisting of a combination of multi‐head self‐attention and a feedforward network—with each token translated into a representative vector of dimension *N* ^hidden^. Specifically, for token *k*(≥ 1), the attention block at layer *l*(1 ≤ *l* ≤ *N* ^layer^) produces the representative vector $h_{l}^{k}\in R^{N^{\mathrm{hidden}}}$. In this formulation, the representative vector *h_t_* at layer *t* is regarded as the internal state at time *t*, i.e., $x\left(t\right)≔h_{t}$. When an input text is represented as a token sequence of length *N* ^token^, *x*(*t*) corresponds to the vector $h_{t}^{1:N^{\mathrm{token}}}$, which is of dimension *N* ^token^ × *N* ^hidden^.

Here, we employed ALBERT^[^ ^37^ ^]^ developed by Google as an encoder‐only model. In ALBERT, since the attention blocks sharing the same parameters are repeatedly applied to the hidden state, it can be strictly interpreted as a dynamical system. In other words, it can be expressed in the form *x* (*t* + 1) =  *F*(*x*(*t*)), and the length of the time series *N* ^iteration^ to be sampled can therefore be freely determined.

*Google/ALBERT*: We used publicly available parameters of the ALBERT *large* model (https://github.com/google‐research/albert), whose hidden dimension *N* ^hidden^ was 1024 and whose total parameter size was 18M (M: million). The token size *N* ^token^ was set to 32, 64, and 128, enabling us to change the dimension of the systems to 32768, 65536, and 131072, respectively. We randomly sampled input sentences from the English Wikipedia dataset, which was initially used for the pretraining of the model. By iteratively applying the layer of the Transformer's encoder, the dynamics of token vectors were obtained for 2 × 10^5^ time steps (*N* ^iteration^ =  2 × 10^5^). Since the representative vectors began to synchronize among tokens after a few hundred‐time steps from the initial input,^[^ ^41^ ^]^ we analyzed the synchronized time‐series dataset of the internal network dynamics, represented as an *N* ^iteration^ × *N* ^hidden^ matrix.

*Decoder‐Only Model*: Decoder‐only models are neural networks composed of *N* ^layer^ attention blocks that generate representative vectors from a token sequence of length *N* ^token^. In contrast to encoder‐only models, decoder‐only models yield the next token $s_{N^{\mathrm{token}}+1}$ using the representative vector of the final layer for the last token, $h_{N^{\mathrm{layer}}}^{N^{\mathrm{token}}}$ (in many cases, the logits for each token are computed with a linear layer $W^{\mathrm{head}}\in R^{\left|V\right|\times N^{\mathrm{hidden}}}$ referred to as a *classification head*). Models that generate tokens in this manner are generally termed *autoregressive* models.

Unlike encoder‐only models, the hidden state corresponding to token *t*, $h_{1:N^{\mathrm{layer}}}^{t}$, can be seen as the internal state at time *t* (i.e., $x\left(t\right)≔h_{1:N^{\mathrm{layer}}}^{t}$). In contrast to ALBERT, decoder‐only models cannot be strictly interpreted as usual dynamical systems, since token generation depends on the internal state—more precisely, on the values of the *key* and *value* within the attention blocks—for all preceding tokens. In other words, they cannot be described in the form *x*(*t* + 1)  =  *F*(*x*(*t*)). However, we refer to the temporal evolution of the internal state as the *dynamics* for consistency with encoder‐only models.

Wte analyzed the dynamics of three models classified as decoder‐only: GPT‐2^[^ ^38^ ^]^ by OpenAI; Llama‐3.1^[^ ^39^ ^]^ by Meta; LLM‐jp‐3,^[^ ^40^ ^]^ a Japanese variant of Llama developed by NII in Japan (https://huggingface.co/llm‐jp/llm‐jp‐3‐13b).

*GPT 2 by OpenAI*: We employed the publicly available GPT‐2 (https://github.com/openai/gpt‐2, parameter size: 124M). GPT‐2 is a decoder‐only model composed of attention blocks with a hidden dimension of *N* ^hidden^ =  768 and a total of *N* ^layer^ =  12 layers ($x\left(t\right)\in R^{N^{\mathrm{layer}}\times N^{\mathrm{hidden}}}$). The context length of GPT‐2 is 1024, allowing it to generate up to 1024 tokens including the input prompt. As the internal state corresponding to the token at the final time step *t*  =  1024 is not computed, a total of 1023 time steps of the time series were sampled and analyzed (*N* ^iteration^ = 2^10^  − 1).

The input prompts were prepared by selecting 200 samples of test data from the WebText dataset, which was originally used for pretraining GPT‐2 (https://github.com/openai/gpt‐2‐output‐dataset). Each input prompt was tokenised using a BPE (Byte‐Pair Encoding) tokenizer and the first 20 tokens of each were extracted as the input prompt. The remaining 1004 tokens were generated by GPT‐2. The next token was selected stochastically based on the probability distribution computed via the final layer's hidden states $h_{N^{\mathrm{layer}}}$ and the subsequent logits $W^{\mathrm{head}}h_{N^{\mathrm{layer}}}$. A fixed random seed was set to ensure reproducibility of the experiments. Moreover, the typical hyperparameters for adjusting the probability distribution—namely temperature, top_k and top_p—were not specified, and the default values (each set to 1) were employed.

*Llama‐3.1 by Meta*: We employed the publicly available Llama‐3.1‐8B (https://github.com/meta‐llama/llama‐models, parameter size: 8B, where B stands for billion; hereafter referred to as Llama‐3.1). Llama‐3.1 is a decoder‐only model composed of attention blocks with a hidden dimension of *N* ^hidden^ =  4096 and a total of *N* ^layer^ =  32 layers. Although Llama‐3.1 has a context length of 131072, the token length was limited to 16384, and the corresponding internal state was analyzed (*N* ^iteration^ = 2^14^  − 1).

The first 20 tokens were extracted from each of the 20 test samples in the WebText dataset. Tokens were generated stochastically under the same settings as for GPT 2 (with a fixed random seed and no specified hyperparameter adjustments). Typically, Llama‐3.1 inserts a special token “<|begin_of_text|>” at the beginning of the input prompt; however, to maintain consistency with the GPT‐2 setup, “<|begin_of_text|>” was omitted. This did not significantly affect the completion quality. In addition, while token generation would normally be terminated upon the generation of the special token “<|end_of_text|>”, generation was continued until the length reached *N* ^iteration^.

*LLM‐jp‐3 by NII*: We also employed the publicly available LLM‐jp 3–13B, a decoder‐only model that was developed by NII, Japan (https://huggingface.co/llm‐jp/llm‐jp‐3‐13b, parameter size: 13B; hereafter referred to as LLM‐jp‐3) and shared its architecture with Llama 3.1 (*N* ^hidden^ =  5120, *N* ^layer^ =  40). LLM‐jp‐3 uses a tokenizer specifically designed for Japanese and is primarily pretrained on multiple Japanese corpora. The context length of LLM‐jp‐3 is 4096 tokens, and the dynamics were sampled up to this limit (*N* ^iteration^ = 2^12^  − 1). From the dataset used for pretraining LLM‐jp‐3 (https://gitlab.llm‐jp.nii.ac.jp/datasets/llm‐jp‐corpus‐v3), 20 validation samples formatted from the Japanese Wikipedia were selected, and the first 20 tokens from each were extracted to serve as the input prompt. As with GPT‐2, a fixed random seed was used, and no hyperparameters were specified. Moreover, as with Llama‐3.1, the special token denoting the beginning of a sentence, “<s>”, was omitted, and the sampling continued even if the special token indicating the end of a sentence, “</s>”, was generated.

*Preprocessing of LLM Data for Energy Landscape Analysis*: As shown in an example displayed in Figure S1a,b (Supporting Information), the internal dynamics in the LLMs were likely to lose the fluctuation after 10^5^ iterations. Therefore, to reduce the effects of such relatively stable network activity, we used the data before 6×10^4^ iterations. In addition, we also excluded the first 10^4^ iterations to decrease the confounding effects of the sample sentences input into the LLMs as an initial condition. In sum, we analyzes the data obtained between 10^4^ and 6×10^4^ iterations (i.e., the data in the red box in Figure S1a, Supporting Information).

We then performed temporal normalization for each activity recorded from each node and conducted *k*‐means clustering to classify the *N* ^hidden^ nodes into nine clusters (Figure S1c, Supporting Information). We set the number of clusters at nine since the whole‐brain neural activity was also represented as time‐series data of the nine large‐scale brain networks.

Based on the *k*‐means clustering, we calculated the mean activity for each cluster and binarized the data for the following energy landscape analysis. We set the threshold for the binarization at the temporal average of each cluster activity.

As a result of these procedures, an activity pattern of the nine clusters at time point *t* was denoted as $V^{t}=\left[\sigma_{1}^{t},\sigma_{2}^{t},\text{…},\sigma_{N}^{t}\right]$, where $\sigma_{i}^{t}$ represented a binary activity of cluster *i* at time *t* (i.e., $\sigma_{i}^{t}=+1\;\mathrm{or}-1$) and *N* represents the number of clusters (i.e., *N* = 9).

We prepared such preprocessed binary data for each of the LLM internal dynamics.

For the decoder‐only models, we performed this preprocessing and the following energy landscape analysis for each layer separately. Afterward, we calculated the two Gini coefficients and estimated the averages of the two coefficients across layers. Note that we excluded the data of the first layer since the internal states in the first layer merely represent input tokens.

### Energy Landscape Analysis

For both the brain data and LLM data, we applied the same energy landscape analysis to the binarized time‐series data (i.e., $V^{t}=\left[\sigma_{1}^{t},\sigma_{2}^{t},\text{…},\sigma_{N}^{t}\right]$) in the same manner as in the previous studies.^[^ ^16^, ^18^, ^19^, ^23^ ^]^

*Fitting of a Pairwise Maximum Entropy Model*: We fitted a pairwise maximum entropy model (MEM) to the binary data. The MEM consisted of two parameters, *h_i_* and *J_ij_*. *h_i_* is considered to indicate the basal activity of node *i* (here, network *i* for the brain data and cluster *i* for the LLM data), whereas *J_ij_* should represent a pairwise interaction between nodes *i* and *j*.

We determined the two types of parameters so that the average of the MEM‐based node activity 〈σ_*i*_〉_m_ and the average of the MEM‐based pairwise interactions 〈σ_*i*_σ_*j*_〉_m_ are close enough to the average of the empirical node activity 〈σ_*i*_〉 and the average of the empirical pairwise interaction 〈σ_*i*_σ_*j*_〉, respectively. We defined the 〈σ_*i*_〉_m_ as $\Sigma_{\ell =1}^{2^{N}}\sigma_{i}\left(V_{\ell }\right)P\left(V_{\ell }\right)$ and the 〈σ_*i*_σ_*j*_〉_m_ as $\Sigma_{\ell =1}^{2^{N}}\sigma_{i}\left(V_{\ell }\right)\sigma_{j}\left(V_{\ell }\right)P\left(V_{\ell }\right)$, where σ_*i*_(*V_k_*) represented the activity of node *i* in the activity pattern *V_k_* and *P*(*V_k_*) denoted the appearance probability of the neural activity pattern *V_k_*. The *P*(*V_k_*) was given as $e^{-E(V_{k})}/\Sigma_{\ell =1}^{2^{N}}e^{-E(V_{\ell })}$, where $E\;\left(V_{k}\right)=-\Sigma_{i=1}^{N}h_{i}\sigma_{i}\left(V_{k}\right)-\left(1/2\right)\Sigma_{i=1}^{N}\Sigma_{j=1}^{N}J_{ij}\sigma_{i}\left(V_{k}\right)\sigma_{j}\left(V_{k}\right).$ In this setting, we adjusted *h_i_* and *J_ij_* until the 〈σ_*i*_〉_m_ and 〈σ_*i*_σ_*j*_〉_m_ were approximately equal to the 〈σ_*i*_〉 and 〈σ_*i*_σ_*j*_〉 with a gradient ascent algorithm. The fitting accuracy, *r_D_*, was evaluated based on a proportion of Kullback–Leibler divergence in this 2nd‐order model (*D*_2_) to that in the 1st‐order model (*D*_1_)^[^ ^16^, ^17^, ^18^, ^19^, ^23^ ^]^ (i.e., *r_D_* = (*D*_1_ – *D*_2_)/*D*_1_). Here, the 1st and 2nd‐order models indicate independent and pairwise MEM, respectively.

*Energy Landscape Structure*: Using the *h_i_* and *J_ij_*, we determined an energy landscape structure for each individual and each LLM trial. Note that, in the landscape, two activity patterns were regarded as adjacent if and only if their difference was seen at only one node activity.

First, we identified local energy minima, whose energy values were smaller than those of the *N* adjacent activity patterns. We then clarified the hierarchical structures between the local minima by building a dendrogram—a so‐called disconnectivity graph—as follows.^[^ ^16^, ^18^, ^19^, ^23^ ^]^ i) We prepared a hypercube graph in which each vertex represented an activity pattern, *V_k_*, and was adjacent to the *N* neighboring vertices. ii) We set a threshold energy level, *E*_threshold_, at the largest energy value among the 2*^N^* vertices. iii) We then removed the vertices whose energy values *E*(*V_k_*) were greater than *E*_threshold_. iv) We examined whether each pair of local minima remained connected by a path in the slightly disconnected graph. v) We repeated steps (iii) and (iv) after changing *E*_threshold_ down to the next largest energy value. vi) We stopped these procedures when all the remaining local minima were isolated. vii) Based on the obtained results, we built a hierarchical tree whose leaves (i.e., terminal vertex down in the tree) represented the local minima and internal vertices indicated the branching points of different local minima.

*Classification to Attractors*: Based on this individual disconnectivity graph, we classified all the activity patterns *V_k_* (*k* = 1, 2, …, 2*^N^*) into one of the attractors with a corresponding local minimum at their bottoms. First, we picked up an activity pattern *V_i_* from the 2*^N^* patterns. If any of its neighbor patterns had a smaller energy value than *V_i_*, we moved to such an activity pattern. Otherwise, we did not move because the *V_i_* was a local minimum. We repeated this procedure for all the *V_i_* until reached any of the local minima. The initial *V_i_* was then classified into a member of the basin of the local minimum that we finally reached. Through this procedure, we classified all the activity patterns on the energy landscape—except for nodes on the saddles—into any of the attractors.

*Random Walk Simulation*: Using these results, we performed a random‐walk simulation and estimated state dynamics on the energy landscape. The simulation was based on a Markov chain Monte Carlo method with the Metropolis–Hastings algorithm.^[^ ^74^, ^75^ ^]^

In this simulation, an activity pattern *V_i_* moved only to a neighboring pattern *V_j_*. First, we randomly chose one of such neighboring patterns and then determined whether such a movement to the neighbor occurred or not at the probability $P_{ij}=\min\left[1,e^{E\left(V_{i}\right)-E\left(V_{j}\right)}\right]$. In other words, when *V_i_* was more unstable than *V_j_* (i.e., *E*(*V_i_*) > *E*(*V_j_*)), the activity pattern always moved from *V_i_* to *V_j_*. In the meantime, this setting left some room for moving to *V_j_* even if *V_i_* was more stable than *V_j_* (i.e., *E*(*V_i_*) < *E*(*V_j_*)), which prevented the activity pattern from being stuck in a local minimum forever.

For each individual or LLM trial, we repeated this random walk 10^5^ steps with a random initial pattern, which resulted in a trajectory of the activity pattern such as $\left[V^{1},V^{2},\text{…},V^{10^{5}}\right]$. After discarding the first 100 steps to reduce the effects of the initial condition, we classified all the *V^t^* into either of the attractors and converted $\left[V^{101},V^{102},\text{…},V^{10^{5}}\right]$ to, for example, [Attractor #1, Attractor #3, Attractor #2, …].

*Calculations of Dwelling Time and Transition Frequency*: Next, we assessed how long each attractor continued in the trajectory (dwelling time) and how often one attractor switched to another attractor in the sequence (transition frequency). This was performed for each individual or each LLM trial.

### Gini Coefficients

Finally, we calculated Gini coefficients—an index for polarization and bimodality—for the dwelling time and transition frequency. We estimated the coefficients for each individual and each LLM data and compared them between different aphasia groups and LLMs.

To infer correlations between this index and aphasic behaviors, we calculated Pearson correlation coefficients i) between Gini coefficients for the dwelling time and fluency scores and ii) between Gini coefficients for the transition frequency and comprehension scores. Both the fluency score and comprehension score were rated based on Western Aphasia Battery.

Given the characteristics of each aphasia, the former correlation was estimated for Broca aphasia, whereas the latter was assessed for the two receptive aphasia (i.e., conduction and Wernicke's aphasia).

### Statistics

The Gini coefficients were compared between the different types of aphasia and controls using two‐sample *t*‐tests. The effects of the multiple comparisons were corrected using Bonferroni's method.

### Data and Code

All the aphasia data were openly available in the repositories: the ARC dataset^[^ ^71^ ^]^ and the PREVENT‐AD project.^[^ ^72^ ^]^ The code for energy landscape analysis is shared as Supporting Information for the previous study.^[^ ^76^ ^]^

### Ethics

We used openly available neuroimaging data, all of which were recorded under the approval of local ethics committees. See Experimental Section for details.

## Conflict of Interest

The authors declare no conflict of interest.

## Author Contributions

T.W., K.N., and K.A. conceived this study. T.W., K.I. and K.N. analyzed the data. T.W., K.I., Y.K., K.N., and K.A. discussed the results. T.W. wrote the manuscript.

## Supporting information



Supporting Information

## Data Availability

All the aphasia data were openly available in the repositories: the ARC dataset and the PREVENT‐AD project.
