# Supplementary material for: Comparison of Large Language Model with Aphasia
Source: Adv Sci (Weinh). 2025 May 14;12(22):2414016. doi: 10.1002/advs.202414016 (PMC12165151; doi:10.1002/advs.202414016)
Supplement: Supplementary file 1 — Supporting Information [file ADVS-12-2414016-s001.docx]

**Supporting Information**


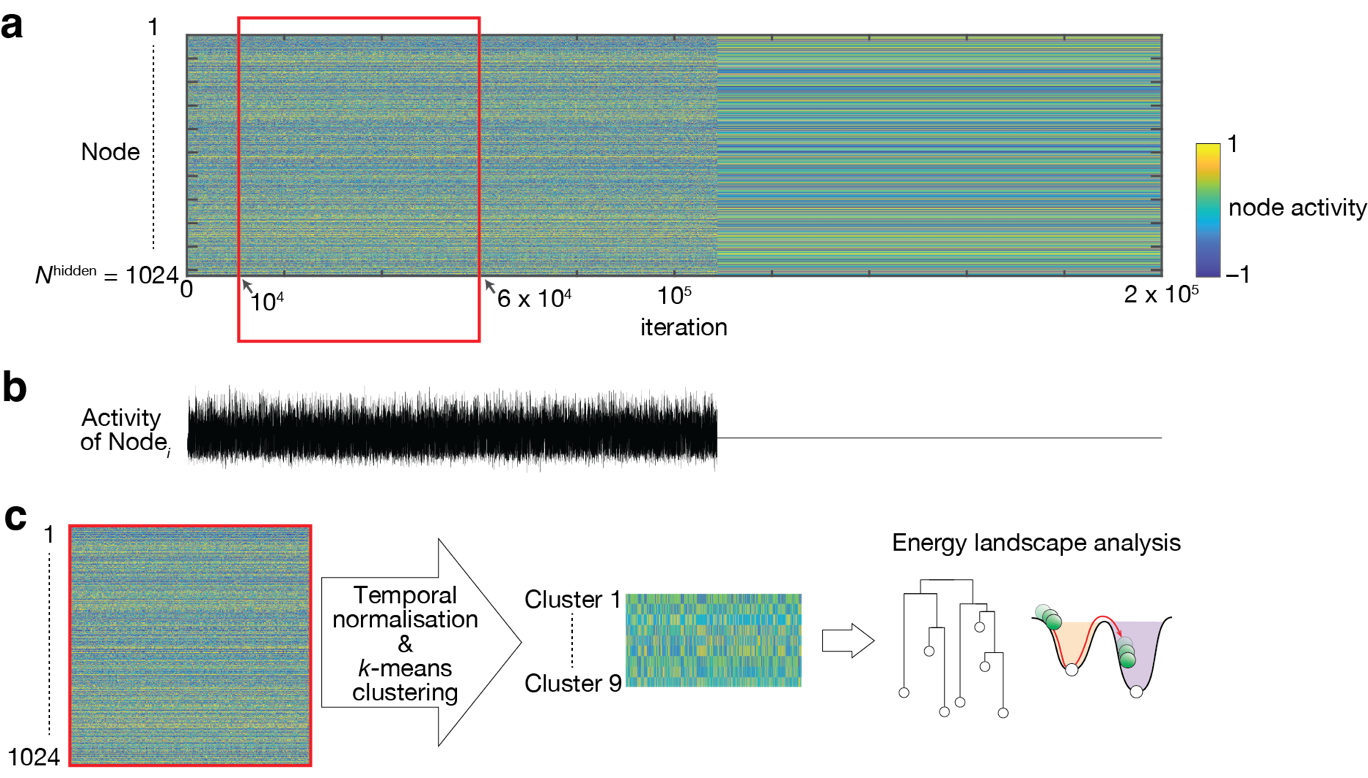


**Supplementary Fig. 1. Preprocessing of LLM data. a.** We first recorded the internal network dynamics that occurred in LLMs as a response to a token. To analyse internal fluctuation, we extracted network dynamics that was recorded between 10^4^ and 6 x 10^4^ iterations, which is indicated as a red box. **b.** The wave indicates an example of the activity of a certain node in the LLM. After certain iterations (e.g., 1.2 x 10^5^ iterations), the node activity lost its fluctuation. **c.** We then applied temporal normalisation and *k*-means clustering to the extracted data. Since the whole-brain activity was summarised into nine time-series data of the nine representative networks, we clustered the LLM data into nine clusters. Finally, we applied energy landscape analysis to this dataset.
